# The first case of SMARCB1 (INI1) - deficient squamous cell carcinoma of the pleura: a case report

**DOI:** 10.1186/s12885-018-4321-x

**Published:** 2018-04-07

**Authors:** Kazushi Yoshida, Yutaka Fujiwara, Yasushi Goto, Takashi Kohno, Akihiko Yoshida, Koji Tsuta, Yuichiro Ohe

**Affiliations:** 1 Department of Thoracic Oncology, National Cancer Center Hospital, 5-1-1, Tsukiji, Chuo-ku, Tokyo, 104-0045 Japan; 2 Division of Genome Biology, National Cancer Center Research Institute, Tokyo, Japan; 3 Department of Pathology and Clinical Laboratories, National Cancer Center Hospital, Tokyo, Japan; 4 Department of Clinical Sciences and Laboratory Medicine, Kansai Medical University, Moriguchi, Japan

**Keywords:** SMARCB1(INI1), Thoracic malignant tumor, Chemotherapy

## Abstract

**Background:**

*SMARCB1 (INI1)* is a tumor-suppressor gene located at 22q11.2. Loss of SMARCB1 protein expression has been reported to be associated with atypical teratoid/rhabdoid tumors and malignant rhabdoid tumors of the kidney and extrarenal tissues. To date, however, SMARCB1-deficient carcinoma of the pleura has not been reported. We report the first case of SMARCB1- deficient squamous cell carcinoma of the pleura.

**Case presentation:**

The case was a 33-year-old female. She was diagnosed squamous cell carcinoma of the pleura by thoracoscopy. The tumor cells were completely negative for SMARCB1 protein expression by immunohistochemistry. She received six cycles of cisplatin plus gemcitabine therapy and TS-1 monotherapy, however, her disease progressed rapidly with worsening chest pain and dyspnea, and she died at 10 months after diagnosis.

**Conclusions:**

This is the first report of SMARCB1-deficient squamous cell carcinoma of pleura. The tumor was highly aggressive and carried a poor prognosis with short survival. The clinical features and treatments of this tumor are not clear, and additional cases will assist the establishment of treatments.

## Background

*SMARCB1 (INI1)* is a tumor-suppressor gene located at 22q11.2. It is considered as an integral component of the chromatin remodeling complex SWI/SNF [1], but the function is largely unknown. Loss of SMARCB1 expression has been reported to be associated with atypical teratoid / rhabdoid tumors (AT / RT) and malignant rhabdoid tumors (MRTs) of the kidney and extrarenal tissues [2]. Furthermore sinonasal basaloid carcinomas and neoplasms arising from the gastrointestinal tract, pancreas and uterus with SMARCB1 deficiency have been reported. However, SMARCB1- deficient carcinoma of the thoracic cavity including lung and pleura has not been reported. We report the first case of SMARCB1-deficient squamous cell carcinoma of the pleura in a patient.

## Case presentation

**Fig. 1:**
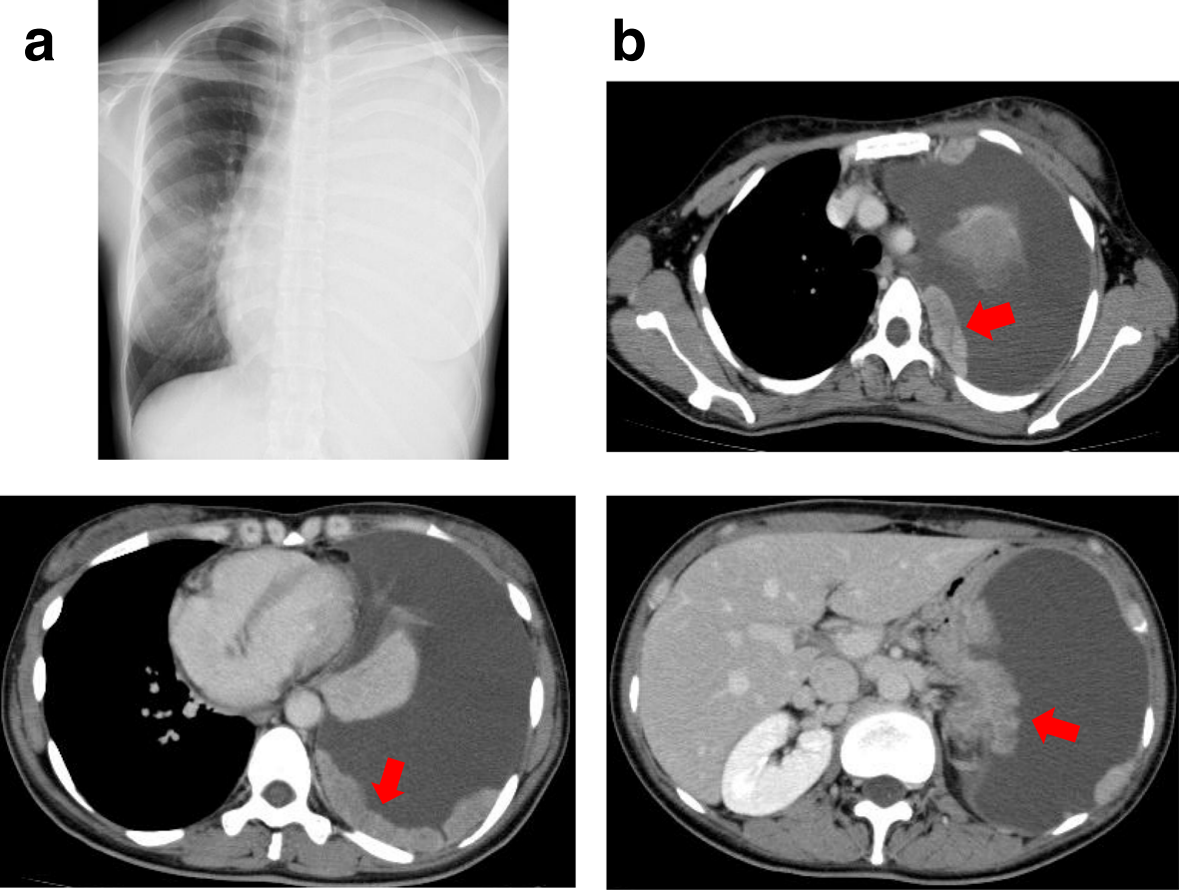
Chest X-ray and Contrast-enhanced computed tomography at the initial diagnosis. **a** Chest x-ray showed a large amount of pleural effusion. **b** Contrast-enhanced computed tomography of the chest-mediastinal window showed multiple irregular pleural thickening (the red arrows) of the left lung with pleural effusion

The case was a 33-year-old female, with no history of smoking, previous medical or family history of malignant disease. She visited the previous hospital with one-month history of worsening cough and dyspnea. Chest X-ray and Contrast-enhanced computed tomography (CT) showed left pleural tumors with a large amount of pleural effusion (Fig. 1).The tumor was limited to the left thoracic cavity, she underwent the diagnostic thoracoscopy to obtain tumor tissue from the parietal pleura sufficient for further analysis. Pathological diagnosis in the previous hospital was squamous cell carcinoma of the pleura. She received six cycles of cisplatin plus gemcitabine therapy. The best response of the chemotherapy was stable disease. After progression, she visited to our institution to be received further treatment. She received TS-1 monotherapy (100 mg/body) as second-line treatment. However her disease progressed rapidly with worsening chest pain and dyspnea, and she died 10 months after diagnosis.

### Histological analysis

**Fig. 2:**
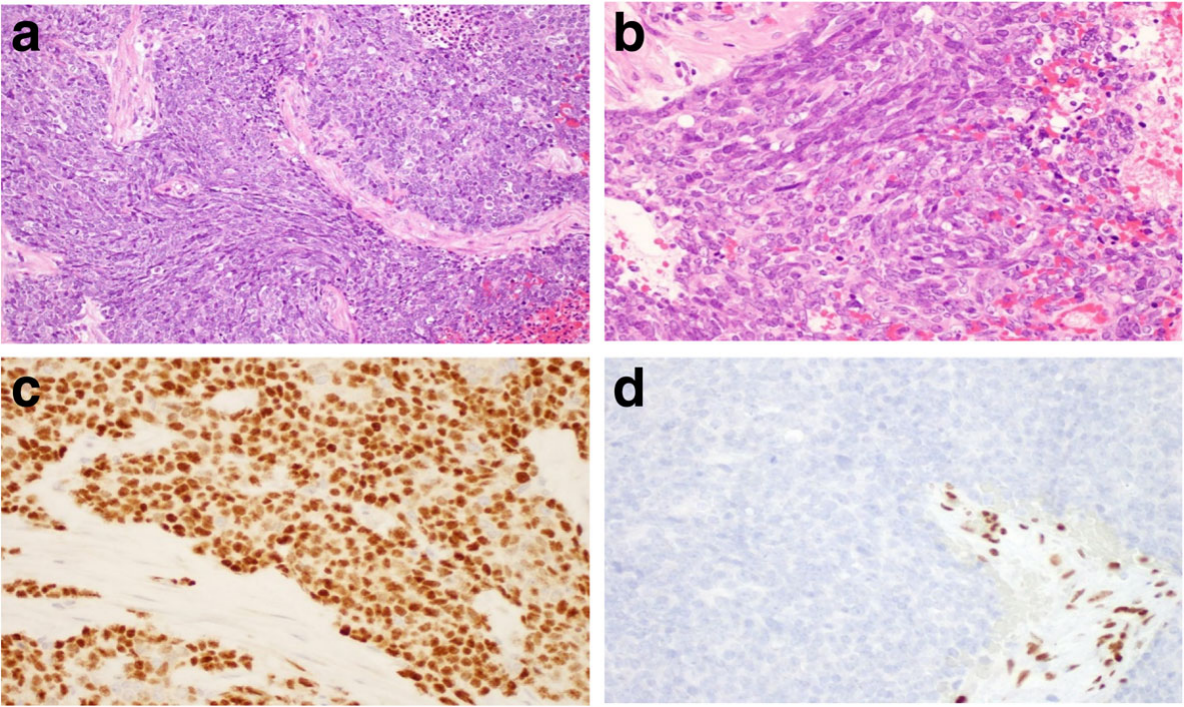
Histopathological findings of the pleural tumor obtained by thoracoscopy. **a, b** Hematoxylin and eosin-stained (HE) shows low differentiated carcinoma that have characteristics of squamous cell carcinoma. **a** was 100X, **b** was 400X magnification. **c** Tumor cells staining positive for p40. **d** Tumor cells staining negative for SMARCB1

Upon histological review of the submitted biopsy slide, the tumor was composed of sheets of poorly differentiated cells with some streaming pattern. The stroma was neither myxoid nor hyalinized, and rhabdoid cells were absent. Immunohistochemically (IHC) staining revealed that the tumor cells were diffusely positive for cytokeratin (CK) 5/6, p40, desmocollin 3, claudin 4, and MOC31, whereas they were negative for WT1, D2-40, S100 protein, GFAP, CD34, c-kit, NUT, and calponin. The results confirmed the diagnosis of non-keratinizing squamous cell carcinoma. An additional IHC unexpectedly revealed the complete lack of SMARCB1 expression (Fig. 2). Given the lack of radiological evidence of tumor involvement of the lung, mediastinum, and head and neck, the disease was clinically considered primary to the pleura.

### Molecular analysis

**Fig. 3:**
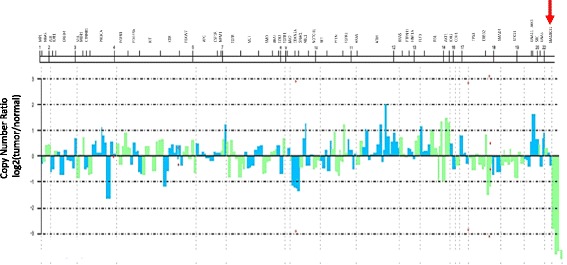
Target sequencing by next-generation sequence by The Ion AmpliSeq™ Cancer Hotspot Panel v2.Red allow indicates copy number loss of *SMARCB1 gene* of this tumor

We analyzed hotspot mutations from the formalin-fixed paraffin-embedded (FFPE) tissues, using the Ion AmpliSeq™ Cancer Hotspot Panel v2 that targeting “mutation-hotspot region” in 50 most common oncogenes. The sequence result showed copy number loss of *SMARCB1* gene in this tumor (Fig. 3). And another next-generation sequencing assay (Agilent SureSelect NCC oncopanel) [3] revealed no other common oncogene mutations, such as epidermal growth factor receptor (EGFR) mutation, anaplastic lymphoma kinase (ALK), RET or ROS1 rearrangements.

## Discussion

We demonstrated squamous cell carcinoma of that pleura which was characterized by SMARCB1 deficiency. *SMARCB1* gene is one of subunits of SWI/SNF complex. SWI/SNF complex links between ATP-dependent chromatin remodeling and DNA repair in several forms. *SMARCB1* is considered as a tumor suppression gene. Specific *SMARCB1* biallelic inactivating mutations were discovered in majority cases of malignant rhabdoid tumors (MRTs) [4]. Thereafter *SMARCB1* gene alternations were identified in other malignant tumors, such as epithelioid sarcoma, myoepithelial carcinoma [5, 6]. Recently, SMARCB1-deficient carcinomas arising from some visceral organs including sinonasal tract, gastrointestinal tract and pancreas have been reported. However, the carcinoma from pleura has never been reported.

The present tumor showed otherwise classic histological features for non-keratinizing squamous cell carcinoma. Specifically, it demonstrated nest and sheet of proliferation with streaming, and it was diffusely positive for CK5/6, p40, and desmocollin 3. Nevertheless, it was clinically atypical in many respects. First, the tumor involved the pleural surface without clear involvement of the lung parenchyma. In addition, CT screening identified no tumors in the mediastinum and other organs, which lead us to interpret this as being primary to the pleura. Pleural-primary squamous cell carcinoma is unheard of exceptional Secondly, the tumor affected a young woman with no history of smoking. This is in sharp contrast to most squamous cell carcinoma of the lung, which typically affects middle-aged and elderly with a strong association with smoking history [7]. In fact, it is these unusual clinical attributes that prompted us to attempt a SMARCB1 staining at original diagnosis.

The differential diagnosis of the present case included malignant mesothelioma, thymic carcinoma, NUT carcinoma, epithelioid sarcoma, malignant rhabdoid tumor, and myoepithelial carcinoma. Pleura-based tumor spread was indeed reminiscent of mesothelioma; however, diffuse expression of p40, MOC31, and claudin 4 and negative expression of WT1 and D2-40 ruled out this possibility. Although most thymic carcinoma takes the form of squamous cell carcinoma, the current tumor did not involve the mediastinum and lacked expression of c-kit, a highly sensitive marker for thymic carcinoma. NUT carcinoma often demonstrates squamous differentiation, but the defining features of this entity, i.e., nuclear NUT protein expression due to NUT gene rearrangement is lacking in the present tumor. Epithelioid sarcoma and malignant rhabdoid tumor are 2 prototypical sarcoma types that are deficient in SMARCB1. These tumors often express CD34 and lack clear-cut epithelial and squamous differentiation. Approximately 10% of soft-tissue myoepitheliomas in adults are known to be deficient in SMARCB1. Myoepithelial and squamous differentiation could partly overlap, as CK5/6 and p40 may be expressed in a small subset of soft-tissue myoepithelial tumors. However, the characteristic myoepithelial histology such as reticular or cord-like growth and hyalinized or myxoid stroma was entirely absent in the present tumor. Moreover, soft-tissue myoepitheliomas are usually positive for S100 protein and/or GFAP, and many cases express calponin, whereas these markers were completely negative in our pleural tumor. [8].

One interesting analogy to our case is with the recently characterized SMARCB1-deificnet sinonasal carcinomas. In a recent series by Agaimy et al. [9] compiling 39 cases, 5 of them were diagnosed non-keratinizing or basaloid squamous cell carcinoma. In IHC analysis, all case (5/5) were positive for CK5 and 60% (3/5) were positive for p63. Furthermore, from the clinical point of view, the age range was diverse (19 to 89 years, median 52 years) in these 39 cases [9], and the link between smoking and cancer development of sinonasal carcinoma is much weaker than other head and neck cancer [10, 11]. Thus, there is a clinicopathological similarity between the present case and SMARCB1-deficient sinonasal carcinoma. Of note, our patient had no evidence of tumor involvement of the sinonasal tract. Whether our case could be understood as a thoracic counterpart of SMARCB1-deficient sinonasal carcinoma remains to be seen based on future studies of a large number of cases.

SMARCB1-deficient tumors frequently have a poor prognosis with wide spread metastasis at the time of diagnosis [12]. Pediatric AT/RT is the most major tumor of SMARCB1 deficiency. The multimodality treatments of AT/RT have been improved, however, AT/RT still carries poor prognosis of the median survival time of 17monthes [13].Likewise, gastrointestinal tract carcinomas with SMARCB1-deficiency progress aggressively with no response for cytotoxic chemotherapy, and their mean survival were only 4 months [14]. 39 cases of SMARCB1-deficient sinonasal carcinoma also have a poor prognosis. A half of them were died of disease 0 to 102 months (median 15 months) after diagnosis. Concordantly, our case was also an aggressive disease which carried a poor prognosis with short survival like other SMARCB1-deficient tumors. Recently, epigenetic approaches have been reported as a therapeutic potential for SMARCB1-deficient tumors. EZH2 is a histone-lysine N-methyltransferase enzyme that contributes to DNA methylation and transcriptional repression. In vivo tumor models, inactivation of EZH2 blocked tumorigenesis driven by SMARCB1 loss, completely [ [15]]. And a preclinical study has reported that EZH2 inhibitors can inhibit MRT cell proliferation efficiently [16]. The EZH2 inhibitors have a possibility of promising drug for SMARCB1-deficient tumors.

## Conclusion

Herein, we reports the first case of SMARCB1-deficient squamous cell carcinoma of the pleura whose prognosis was poor as with another SMARCB1 deficient tumors. Cytotoxic chemotherapies are tended to be resistance for these tumors, however, some new epigenetic approaches may improve the prognosis. To investigate the characteristics and prognosis, further more cases are needed.

## A**bbreviations**

ALK: Anaplastic lymphoma kinase
CK: Cytokeratin
CT: Computed tomography
EGFR: Epidermal growth factor receptor
FFPE: Formalin-fixed paraffin-embedded
IHC: Immunohistochemistry
MRTs: Malignant rhabdoid tumors
